# Supplementary material for: A Novel Heat Shock Transcription Factor (ZmHsf08) Negatively Regulates Salt and Drought Stress Responses in Maize
Source: Int J Mol Sci. 2021 Nov 3;22(21):11922. doi: 10.3390/ijms222111922 (PMC8584904; doi:10.3390/ijms222111922)
Supplement: Supplementary file 1 [file ijms-22-11922-s001.zip › ijms-1407489-supplementary.pdf]

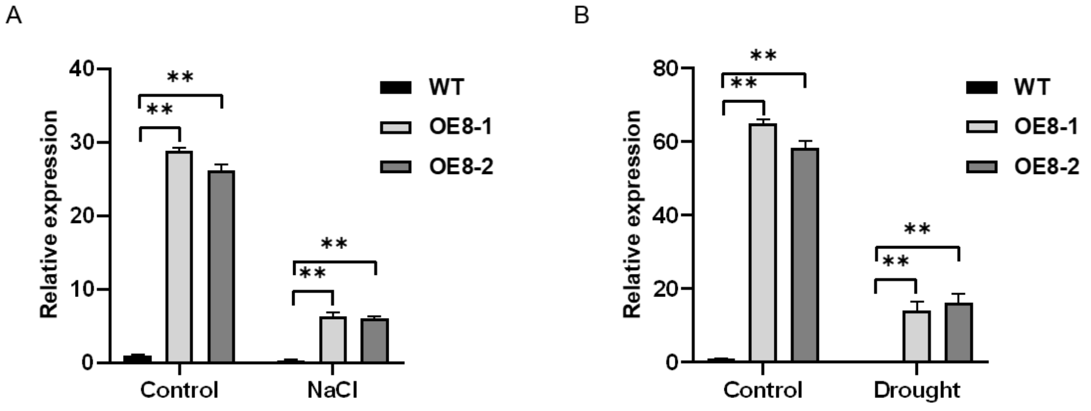

Figure S1: The expression level of *ZmHsf08* in transgenic maize under salt- and drought-stress. A, Relative expression level of *ZmHsf08* in WT and transgenic lines under normal condition and salt treatment for 7 days. B, Relative expression level of *ZmHsf08* in WT and transgenic lines under normal condition and drought treatment for 7 days. Values are means ± SD. Bars represent means ± SD (n = 3 repeats). Significant differences (Student's t test): *, $P < 0.05$, **, $P < 0.01$.

Table S1: Primers for vector construction.

Vector name	Primer names	Primer sequences (5'-3')
ZmHsf08-GFP	GFP-F	GGactagtATGGTGGAGGAAGGCGCC
	GFP-R	CGCggatccATTCCCGCCGCCGCA
GFP-ZmHsf08	DC-F	gatgaactatacaaaggcgcgccAATGGTGGAGGAAGGCGC
	DC-R	cgatcggggaaattcgagctcTTAATTCCCGCCGCCGCA
ZmHsf08-AD	AD-F	gccatggaggccagtgaattcATGGTGGAGGAAGGCGCC
	AD-R	atgcccacccgggtggaattcTTAATTCCCGCCGCCGCA
ZmHsf08-BD	BD-F	atggccatggaggccgaattcATGGTGGAGGAAGGCGCC
	BD-R	tcgacggatccccgggaattcTTAATTCCCGCCGCCGCA
Hsf08-cYFP/nYFP	YFP-F	GCtctagaATGGTGGAGGAAGGCGCC
	YFP-R	CGCggatccATTCCCGCCGCCGCA

Table S2: Primers for RT-qPCR

Gene name	Primer names	Primer sequences (5'-3')
GRMZM2G002131	ZmHsf08-qF	TTGAAGCCTCTGACATCTC
	ZmHsf08-qR	AACCATCACCACCATCTG
GRMZM2G006745	ZmDREB2A-qF	TTGAAGCCTCTGACATCTC
	ZmDREB2A-qR	AACCATCACCACCATCTG
GRMZM2G172230	ZmERD1-qF	CACGATACTGATGGACAT
	ZmERD1-qR	CCTAATCTACTACACTGGAA

GRMZM2G479760	ZmABF2-qF	TTGTGCCTCCACTGCAATTCGG
	ZmABF2-qR	TTACAAGTCGTCTCCCTCCATCTTCC
GRMZM2G098750	ZmRAB18-qF	GTGGTGGGTTGAGGGGAAGGAAGC
	ZmRAB18-qR	ACGCCATCGCCGTTGAGCCT
GRMZM5G838285	ZmNCED-qF	CTTTGAGACTTTCCGAGCAG
	ZmNCED-qR	AGGGAGGATAGGAAGGTAAACA
GRMZM2G379053	ZmZEP-qF	TTGGTTGGTGCCGATGGAAT
	ZmZEP-qR	CGTATCGATATCGGGCAGCA
GRMZM2G704021	ZmLEA2-qF	GTCGAGGACGCGATGTACC
	ZmLEA2-qR	ATTCTCCCCTTGACCGGGAT
GRMZM2G342685	ZmRD20-qF	CTCCTTTGCTGTCCATCCGT
	ZmRD20-qR	TGAGCTCAACTCGTCTTTTGTT
GRMZM2G118019	ZmNHX3-qF	GCCTGGTGTTTGGAGAAGGA
	ZmNHX3-qR	GGACAGTGCTGGTTGCAAAG
